# Systolic Dyssynchrony Index derived from cardiac magnetic resonance imaging predicts left ventricular remodeling in heart failure patients undergoing CRT

**DOI:** 10.1186/1532-429X-13-S1-P175

**Published:** 2011-02-02

**Authors:** Simon G Duckett, Matthew R Ginks, Anoop K Shetty, Matthias Paul, Stam Kapetanakis, Stephen Sinclair, Tobias Schaeffter, C Aldo Rinaldi, Gerry Carr-White, Reza Razavi

**Affiliations:** 1 Kings College London, London, UK; 2 Guy's and St Thomas' Hospital, London, UK

## Introduction

It is known that 30-40% of heart failure patients that undergo cardiac resynchronization therapy (CRT) do not derive significant clinical benefit. Using cine imaging a measure of volume change over the cardiac cycle can be derived called the Systolic Dyssynchrony Index (SDI). This has the potential to improve patient selection pre CRT.

## Purpose

We assessed the potential of the SDI derived from CMR to predict reduction in end systolic volume (ESV) and improvement in ejection fraction in heart failure patients undergoing CRT.

## Method

42 heart failure patients (38 male, ejection fraction 26±8.9%, NYHA 2.8±0.4), 21 with ischemic cardiomyopathy (ICM) and 21 with dilated cardiomyopathy (DCM) underwent a CMR prior to CRT implantation. Patients were scanned using 1.5T MR-scanner (Achieva, Philips Healthcare, Best, Netherlands) with either 32 or 5-element cardiac coil. Cine steady state free precession (cine-SSFP) images of four, three, two chamber as well as a multiple slice short axis stack were acquired (FA=60°, TR/TE=2.9/1.5ms, resolution 2.2x2.2x10mm, 30 heart phases). Using TomTec 4D LV-Analysis MR (TT4DMR) we derived a 16 segment systolic dyssynchrony index (SDI). 2D echo pre and 6 months post CRT implantation was used to assess change in EF and ESV. Patients were considered to have remodeled if there was 15% reduction in ESV. Furthermore ≥ 15% improvement in EF was also considered as responding to CRT. An SDI of 10.3% was used to calculate sensitivity and specificities.

## Results

**Figure 1:**
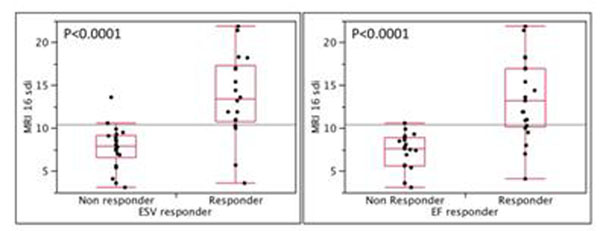
Shows relationship between a percentage SDI and presence of reverse remodeling on the basis of LV end-systolic volume (ESV) (left) and LV EF (right).

All patients had a CRT device and were followed up at 6 months. 19 (45%) of the patients had a reduction in ESV ≥ 15% and 22 (52%) had an improvement in EF of ≥ 15%. There was a highly significant relationship between reduction in ESV and SDI (P<0.0001) as well as a greater than 15% improvement in EF and SDI (P<0.0001) (see figure 1 and table 1). The sensitivity and specificity for CMR derived SDI to predict a 15% reduction in ESV was 0.88 and 0.91 respectively (positive predictive value 0.88, negative predictive value 0.91). The sensitivity and specificity for CMR derived SDI to predict a 15% increase in EF was 0.76 and 0.94 respectively (positive predictive value 0.94, negative predictive value 0.77).

**Table 1:** Shows the change in ESV and EF pre and post CRT

All patients	All patients		Patients with SDI > 10.3		Patients with SDI < 10.3	
	Pre CRT	Post CRT	Pre CRT	Post CRT	Pre CRT	Post CRT

Ejection Fraction (%)	26±8.9	31.8±9.9 P=0.0005	21.7±8.4	34.9±7.8 P<0.0001	28.9±7.9	28.3±7.7 N/S
End Systolic Volume (ml)	171±65	157±66 P=0.03	197±74	145±55 P<0.0001	154±54	167±67 N/S

## Conclusion

SDI derived from CMR is highly predictive at selecting which patients are likely to remodel post CRT. This may be clinically useful to help identify which patients are likely to responders to CRT.

